# Pin1 is related with clinical stage of papillary thyroid carcinoma

**DOI:** 10.1186/s12957-016-0847-z

**Published:** 2016-03-31

**Authors:** Lixin Jiang, Haidi Chu, Haitao Zheng

**Affiliations:** Gastrointestinal Surgery Ward I, Thyroid Surgery Ward, Affiliated Hospital of Qingdao University – Yantai YuHuangDing Hospital, Yantai, Shandong China

**Keywords:** Papillary thyroid carcinoma, Pin1, Immunohistochemistry, Oncogenic signaling pathway, Treatment

## Abstract

**Background:**

The context and aim of this article was to investigate whether the expression level of Pin1 was in association with the clinical stage of papillary thyroid carcinomas.

**Methods:**

Seventy-two patients who had been treated at the Affiliated Hospital of Qingdao University – Yantai YuHuangDing Hospital during January 2013 to December 2014 were rolled in. The expression levels of Pin1 using immunohistochemistry were tested and were divided into four groups according to the different clinical stages and final scores based on multiplying intensity and percentage value of IHC results. Data was analyzed with SPSS 20.0, and *P* value <0.05 had been chosen as significant.

**Results:**

Considered from analysis result, the Pin1 expression status statistically significantly correlated with the PTC clinical stages (*χ*^2^ = 8.128, *P* = 0.043); as the clinical stage proceeded, the intensity of Pin1 in PTC cells had been increased. But we did not find any relationships between immunohistochemical staining results and other clinicopathological characteristics.

**Conclusions:**

The PTC cells’ intensity of Pin1 was in association with the clinical stage. The role played by Pin1 in PTC has been studied, and we need to further investigate the application of Pin1 in the treatment of PTC.

## Background

Thyroid cancer constitutes only 1 % of all epithelial malignancies worldwide, and it is the most frequent endocrine neoplasms [1]; at present, the incidence of thyroid cancer continues to increase, and papillary thyroid cancer (PTC) is the most common histological type of thyroid malignance [2]. The majority of thyroid cancers has a good prognosis after appropriate treatment including surgical procedure, adjuvant radioactive iodine, and TSH suppression therapy. However, the recurrence rate of differentiated thyroid cancer increased up to 30 %, and the death rate was 8 % after initial treatment at 30 years of follow-up [3, 4].

Pin1 is a peptidyl prolyl cis-trans isomerase that specifically binds to the phosphoserine-proline or phosphothreonine-proline motifs of numerous proteins [5]. Pin1 is vigorously overexpressed in diverse of human cancers, such as breast and prostate cancer [6, 7], required for activity and cross-talk of oncogenic pathways [8]. It has also been linked to several other diseases that include Alzheimer’s disease and asthma [9].

It has been well-known that Pin1, acting as an amplifier of phosphorylation signals, regulates a great deal of cell functions, mainly through biological processes such as cell cycle control, centrosome amplification, chromosome instability, transcriptional regulation, RNA processing, cell proliferation and differentiation, and various pathological conditions, and may correlate with poor clinical outcomes [10–12]. Simultaneously, Pin1 has been shown to catalyze substrate dephosphorylation [13–15], regulate protein stability and ubiquitination [16, 17], and influence cellular localization of its targets in vivo [15, 18].

There are some pharmacological experiments which showed inhibition of Pin1 in vitro triggered apoptosis or suppressed the gene expression [19, 20]. All those outcomes indicated that the Pin1 gene plays an oncogenic role in tumorigenesis, and we could make use of it for cancer treatment.

Previous researches have rarely been done to investigate the relationship between Pin1 and papillary thyroid carcinomas (PTCs); Masahiro et al. [21] confirmed Pin1 mRNA expression in PTC by RT-PCR in their investigation, and they pointed out that Pin1 immunoreactivity was significantly higher in PTC than in follicular adenomas (FAs), the same with Andrzej et al. study [22]. In our study, we investigated whether the expression pattern of Pin1 was different in distinct PTC stage.

## Methods

### Patients

Seventy-two participants who had been treated at the Affiliated Hospital of Qingdao University – Yantai YuHuangDing Hospital from January 2014 to December 2014 were enrolled if they met the following criteria: I: had settled in Yantai, Shandong Province, permanently, suffered from PTC, and untreated before (such as drugs, surgeries); II: finished the clinical examinations including ultrasonography of the thyroid and neck; III: undergone thyroid surgery by the same operation team; IV: revealed that all malignant tumors were PTCs by postoperative histopathological analysis; V: excluded patients with metastatic disease (M1) and staged 72 patients according to the 2014 NCCN Guidelines.

### Methods

#### Immunohistochemistry

The expression levels of Pin1 were detected by immunohistochemistry.

The 3-μm-thick sections of formalin-fixed, paraffin-embedded tissues of PTC specimens were placed on slides and then tested. The expression of Pin1 was detected by Pin1 antibody rabbit polyclonal IgG (sc-15340; Santa Cruz Biotechnologies, Santa Cruz, CA, USA). The sections were processed for immunohistochemistry by deparaffinization in Xylene and dehydration through graded alcohols. Antigen retrieval was done in citrate buffer pH 6 by heating the sections at 500 W for 20 min in a microwave oven. The slides were taken out and left it to cool and then washed by TBS. Endogenous peroxidase activity was blocked with 3 % H_2_O_2_ for 10 min. After being washed by TBS ,the serial sections were covered by 3 % BSA at 37 °C in a humid chamber for 1 h and then incubated overnight in the humid chamber, at 4 °C with primary antibodies for Pin1 (1:100). The slides were then briefly washed with TBS and incubated with polymer-based EnVisionTM (ZB-2301 and peroxidase-conjugated affinipure goat anti-rabbit IgG (H + L) ,ZSGB-BIO) for 1 h at 37 °C in the humid chamber. The chromogenic visualization reaction was done by diaminobenzidine (DAB, ZSGB-BIO), counterstained with hematoxylin, mounted, and then examined by a light microscope (Leica Mcrosystems, Wetzlar GmbH). We did not choose positive control; while in the negative control, primary antibody was replaced by TBS.

#### Evaluation of immunohistochemical staining

In the present study, we scored the number of PTC-positive immunostained cells out of 500 in 5 random high-power fields (Leica Mcrosystems, Wetzlar GmbH, ×400); the score criterion was described as follows: protein expression was first semi-quantified by the intensity of staining: 0 when negative staining if there is total absence; 1 when mild; 2 when moderate, and 3 when intense positive staining. Positive PTC cells were scored based on nucleus and cytoplasm staining of Pin1 protein. Pin1 expression was classified semi-quantitatively using the following criteria: 0 when <5 % of PTC cells expressed Pin1 in the nucleus and cytoplasm; 1 when ≥5 to <25 %; 2 when ≥25 % to <50 %; and 3 when ≥50 %. Resulting score was calculated by multiplying intensity and percentage value, and the final score of immunohistochemical (IHC) staining ranged from 0 to 9. Based on multiplication score, the evaluation standard was reformed: 0 (negative; IHC score 0), 1 (mild; IHC score 1–3), 2 (moderate; IHC score 4–6), or 3 (intense; IHC score 7–9).

### Data analysis

Statistical analysis was performed by SPSS 20.0 statistical software. The Kruskal-Wallis Test was performed for determining the association between Pin1 protein expression and PTC clinical stage.

We tried to figure out whether influence was exited with clinicopathological characteristics (age, gender, family history, muscle invasion, diameter of cancer, and lymph node metastasis) and Pin1 expression, and Mann–Whitney U Test was used to make an access.

The results were considered statistically significant if *P* values were <0.05.

## Results

In the current study, we performed immunohistochemistry to examine Pin1 expression in PTC tissues. Clinicopathological features of 72 PTC patients are described in Table 1, and the Pin1 expression details are described in Tables 2, 3, 4, 5, 6, 7, and 8.

**Table 1:** Clinicopathological features of 72 PTC patients in this study

Variables	Number
Age (years)	
<45	29
≥45	43
Gender	
Female	54
Male	18
Family history	
Yes	4
No	68
Muscle invasion	
Yes	10
No	62
Tumor size (cm)	
≤1	27
>1	45
Lymph node metastasis	
Yes	36
No	36
Clinical stage	
I	47
II	3
III	16
IVA	6

**Table 2:** Pin1 expression details with different clinical stages

Group	I	II	III	IVA	
Negative	2	0	0	0	*χ* ^2^ = 8.128
Mild	18	0	5	1	df = 3
Moderate	18	1	4	1	*P* = 0.043
Intense	9	2	7	4	
Total	47	3	16	6	72

**Table 3:** Pin1 expression details with age

Group	Negative	Mild	Moderate	Intense	Total	*P* value
<45	0	11	7	11	29	0.725
≥45	2	12	17	12	43

**Table 4:** Pin1 expression details with gender

Group	Negative	Mild	Moderate	Intense	Total	*P* value
Male	0	7	5	6	18	0.94
Female	2	16	19	17	54

**Table 5:** Pin1 expression details with family history

T						
Group	Negative	Mild	Moderate	Intense	Total	*P* value
Yes	0	1	2	1	4	0.917
No	2	22	22	22	68

**Table 6:** Pin1 expression details with muscle invasion

Group	Negative	Mild	Moderate	Intense	Total	*P* value
Yes	0	6	2	2	10	0.149
No	2	17	22	21	62

**Table 7:** Pin1 expression details with diameter of cancer

Group	Negative	Mild	Moderate	Intense	Total	*P* value
≤1 cm	1	7	8	11	27	0.297
>1 cm	1	16	16	12	45

**Table 8:** Pin1 expression details with lymph node metastasis

Group	Negative	Mild	Moderate	Intense	Total	*P* value
Yes	2	8	15	11	36	0.682
No	0	15	9	12	36	

All patients had taken operation and confirmed with PTC by histopathological analysis; written consents were gotten from all patients.

As we can see in Table 1, there were a total of 72 patients enrolled in this investigation, 29 (40.28 %) were <45 years old and 43 (59.72 %) were ≥45 years; 54 (75 %) females versus 18 (25 %) males; 4 (5.56 %) with family history and 68 (94.44 %) without; 10 (13.89 %) cases of muscle invasion and 62 (86.11 %) not; micro-PTCs (diameter ≤1 cm) were found in 27 (37.5 %) cases, and 45 (62.5 %) were >1 cm; 36 (50 %) patients presented lymph node metastasis, and 36 (50 %) did not.

**Fig. 1:**
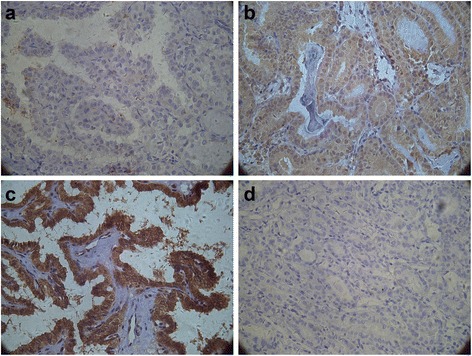
**a**–**d** Immunohistochemical staining present in the expression of Pin1 in different PTC tissues. Pin1 immunohistochemical staining of formalin-fixed, paraffin-embedded tissue sections of PTC tissues(Leica Mcrosystems, Wetzlar GmbH, ×400). **a** Sections of PTCs showing mild immunoreactivity of Pin1 protein expression. **b** Immunoreactivity of Pin1 protein expression was moderate in sections of PTCs. **c** Immunoreactivity of Pin1 protein expression showing intense positive staining in PTC tissues. **d** Pin1 expression was not observed in PTC sections

According to the 2014 NCCN Guidelines, 72 patients were divided into four groups on the basis of their clinical data: 47 patients (65.28 %) had stage I disease, 3 (4.12 %) had stage II disease, 16 (22.22 %) had stage III disease, and 6 (8.33 %) had stage IVA disease. The correlation of immunohistochemical staining results with clinical stages is shown in Table 2, with other clinicopathological characteristics (age, gender, family history, muscle invasion, diameter of cancer, and lymph node metastasis) shown in Tables 3, 4, 5, 6, 7, and 8. The results showed that 70 (97.22 %) PTC sections were positively stained and 2 (2.78 %) were negatively stained. Of the 72 cases, the Pin1 expression was negative (Fig. 1d) in 2 (2.78 %), mild (Fig. 1a) in 24 (33.33 %), moderate (Fig. 1b) in 24 (33.33 %), and intense (Fig. 1c) in 22 (30.56 %); the statistical analysis showed *χ*^2^ = 8.128 and *P* = 0.043. Therefore, the expression of Pin1 was positively correlated with clinical stage in PTCs. We suggested that with the clinical stage proceeding, the intensity of Pin1 in PTC cells had been increased. But we did not find any relationships between immunohistochemical staining results and age, gender, family history, muscle invasion, diameter of cancer, and lymph node metastasis.

## Discussion

There have been few published articles about Pin1 expression in thyroid carcinoma, especially in PTC. In our investigation, we believed this is the first published article about Pin1 expression status specialized in PTC through IHC method, and interestingly, we find Pin1 is believed to have statistically significantly correlated with the PTC clinical stages.

Although there have been some opposed voices that the Pin1 promoter polymorphism is not associated with risk of cancer, indicating that this polymorphism is not a biomarker for susceptibility to cancer [23], we believed identification of Pin1 that were differentially expressed in diverse PTC specimens may be important to predict patient’s prognosis and to develop novel therapeutic strategies for individual treatment.

In the present study, we investigated the expressions of Pin1 in PTCs. The grades of Pin1 expressions are discriminated and scored in PTCs, as we can see in our results. According to the results, we showed that Pin1 upregulation was associated with advanced stage in PTC, but not with other clinicopathological characteristics (age, gender, family history, muscle invasion, diameter of cancer, and lymph node metastasis). Pin1 is believed to have statistically significantly correlated with the PTC clinical stages. There is a similar result worked on esophageal squamous cell carcinoma which had been published by Lin et al. They had explained that Pin1 positively regulated β-catenin and cyclin D1, and they proved that knockdown Pin1 can inhibit aggressiveness of esophageal squamous cell carcinoma (ESCC) cells [24].

It had been reported that Pin1 was not an oncogene itself, but it can serve as a translator and amplifier; play a crucial role in the process of transforming an oncogene to the signal of cell proliferation, differentiation, and apoptosis; and is upregulated in various types of tumor tissues [11].

In Masahiro et al. reports, strong correlation between Pin1 and cyclin D1 immunoexpression and/or cyclin D1 mRNA and Pin1 expression via interaction with Wnt signaling pathway has been observed, and it has been suggested that Pin1 may promote cyclin D1 overexpression directly or through accumulation of beta-catenin in thyroid cancer cells [21].

Andrzej et al. [22] found statistically significant differences in expression of Pin1 messenger RNA (mRNA) between PTC group and benign thyroid lesions (FA, NG), with real-time relative quantification PCR assay for Pin1 mRNA expression, and they have confirmed overexpression of Pin1 gene in PTC. Moreover, no correlations were found between Pin1 expression level and patients’ sex, age, or tumor size in the PTC group, which is the same with our results.

Pin1 catalyzes cis-to-trans conformational switches of the phospho Ser/Thr-Pro (pS/T- Pro) motif in proteins, facilitating kinds of signaling pathways which potentates multiple oncogenic signaling pathways during carcinogenesis [25–27]. It regulates many proteins and factors which are necessary for the mitosis of normal cells [19, 27–29]. However, the overexpression of Pin1 can amplify multiple oncogenic signaling pathways, including Ras [30] and cytokine NF-κB [31]. As a result of its interactions with so many protein and factors, Pin1 plays a complicated role in the regulatory and phosphoproteomic networks [32]. With no surprise, the increased activity of Pin1 is closely associated with many kinds of cancers as mentioned above [6, 7].

Thanks to Pin1’s complicated networks with so many pathways in plenty of cancer cells, drug companies such as Pfizer Global R&D [33–35] and Vernalis Ltd [36, 37] as well as several academic laboratories [38–42] have developed some anti-cancer therapy targeting Pin1. Yoon et al. had suggested Pin1 could be developed as a major therapeutic target in many skeletal diseases [5]. In this article, we also consider its inactivation constituting a promising therapeutic strategy for cancer patients, and we believed that targeting Pin1 pathway could represent a novel modality for treating PTC patients.

There were still some defects in our clinical research, some improvement measures needed to be made, such as enlarge the studies scale in polycentric hospitals and standardized treatment methods. Investigations of the gene-environmental interaction may give us a better understanding of the roles of Pin1 polymorphisms in human cancers.

## Conclusions

In the present study, Pin1 upregulation was suggested to be associated with advanced stage in PTC but not with other clinicopathological characteristics (age, gender, family history, muscle invasion, diameter of cancer, and lymph node metastasis). Pin1 is believed to have statistically significantly correlated with the PTC clinical stages. Further study is required for understanding its pathogenesis, and determining the precise role the Pin1 plays in PTC cell cycle proceeding, which could have important meaning in the diagnostics and treatments in PTCs.

### Ethics approval and consent to participate

This study was reviewed and approved by the hospital ethical committee of patients thyroid carcinomas tissues for our immunohistochemistry (reference number Yantai YuHuangDing Hospital ethical committee,【2015】 number 130), and required informed consent from each patient for use of individual data profiles. Each patient agreed to participate and signed the informed consent form and we excluded patients who were not willing to take part in this experience.

### Availability of data and materials

The dataset(s) supporting the conclusions of this article is(are) available in the e-mail addresses.

## Abbreviations

FAs: follicular adenomas
IHC: immunohistochemical
NG: nodular goiter
PTCs: papillary thyroid cancers
